# The Decade of Super-Resolution Microscopy of the Presynapse

**DOI:** 10.3389/fnsyn.2020.00032

**Published:** 2020-08-11

**Authors:** Georgii Nosov, Martin Kahms, Jurgen Klingauf

**Affiliations:** ^1^Institute of Medical Physics and Biophysics, University of Münster, Münster, Germany; ^2^CIM-IMPRS Graduate Program in Münster, Münster, Germany

**Keywords:** presynapse, active zone, exo-endocytosis, nanoscopy, CLEM

## Abstract

The presynaptic compartment of the chemical synapse is a small, yet extremely complex structure. Considering its size, most methods of optical microscopy are not able to resolve its nanoarchitecture and dynamics. Thus, its ultrastructure could only be studied by electron microscopy. In the last decade, new methods of optical superresolution microscopy have emerged allowing the study of cellular structures and processes at the nanometer scale. While this is a welcome addition to the experimental arsenal, it has necessitated careful analysis and interpretation to ensure the data obtained remains artifact-free. In this article we review the application of nanoscopic techniques to the study of the synapse and the progress made over the last decade with a particular focus on the presynapse. We find to our surprise that progress has been limited, calling for imaging techniques and probes that allow dense labeling, multiplexing, longer imaging times, higher temporal resolution, while at least maintaining the spatial resolution achieved thus far.

## Introduction

The classical chemical synapse in the central nervous system (CNS) of vertebrates is a discontinuous structure consisting of a presynapse formed by the signal transducing neuron and a postsynapse formed by the receiving neuron. The existence of the synapse was originally put forward by Ramón y Cajal (1904), but the first direct observation and most of our current knowledge about the structure of this intercellular contact site has been derived from electron microscopy (EM) (for review see, e.g., Siksou et al., 2009; Harris and Weinberg, 2012). The two halves of the synapse are separated by a synaptic cleft with a width of approximately 15–20 nm (De Robertis and Bennett, 1955; Palay and Palade, 1955) and the presynaptic swelling or bouton is densely filled with granular structures designated as synaptic vesicles (SVs). The discovery of SVs occurred in parallel with establishment of the quantal hypothesis of neurotransmitter (NT) release (Del Castillo and Katz, 1954) and it was only later that SVs were unambiguously identified as subcellular compartments releasing discrete packages of NT (“quanta”) upon fusion with the plasma membrane (PM) (De Robertis et al., 1963; Heuser and Reese, 1973).

The advent of nanoscopic light microscopy techniques more than a decade ago, held the particular promise that nanometer resolution in combination with highly efficient protein labeling strategies, either by immunostaining or genetically encoded fluorescent proteins will greatly increase our understanding of the presynaptic nano-architecture and protein networks far beyond the electron-microscopic picture. Thus, in combination with live cell experiments, nanoscopic light microscopy should contribute to a better understanding of fundamental presynaptic processes such as SV release, compensatory endocytosis and cargo sorting. After briefly summarizing the previous results made by EM, we ascertain the advances in our understanding of the presynaptic nano-architecture driven by the application of nanoscopic techniques.

## The Synapse in the Electron Microscopic Picture

The classical chemical synapse in the vertebrate CNS has a size of 0.5 to 2 μm and can harbor between 100 and 400 SVs in boutons of hippocampal pyramidal neurons. In contrast, large mossy fiber boutons of dentate gyrus granular cells in the hippocampus contain up to several thousand SVs (Schikorski and Stevens, 1997; Rollenhagen et al., 2007). In most mature synapses SVs exhibit a low size variation with a typical diameter of 40–50 nm (De Robertis and Bennett, 1955; Harris and Sultan, 1995). A small pool of SVs is docked at the presynaptic PM at the active zone (AZ), a spatially defined region where SV fusion and NT release occur (Couteaux and Pecot-Dechavassine, 1970; Heuser et al., 1979). Docked SVs are associated with a dense amorphous material, termed the cytomatrix of the presynaptic active zone (CAZ) (Pfenninger et al., 1972; Harlow et al., 2001). In the postsynapse, a submembrane layer of electron-dense material can be distinguished, the so-called postsynaptic density (PSD) (Palay, 1958; Gulley and Reese, 1981). Based on the observation that SVs in the presynapse tend to cluster opposite to the PSD, it is common sense today that the PSD constitutes a postsynaptic cytoskeleton involved in organizing postsynaptic receptor localization face to face to the presynaptic AZ. The size of the average presynaptic AZ, estimated by the size of the PSD is 200–400 nm in diameter (Cohen and Siekevitz, 1978).

**FIGURE 1:**
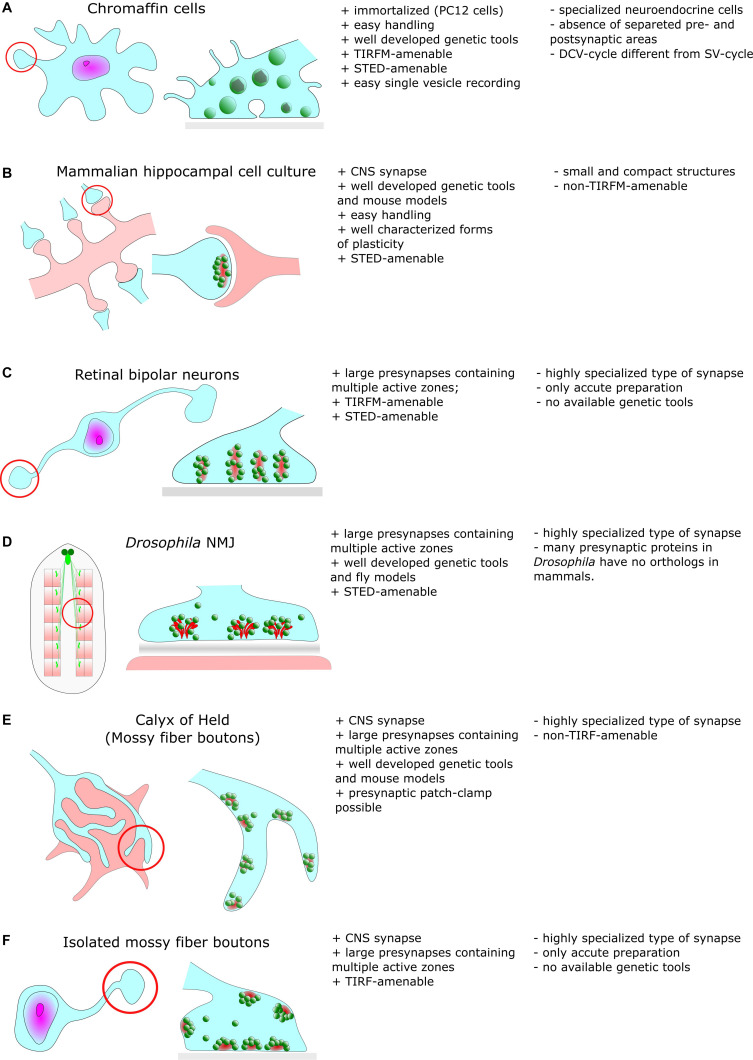
Common cellular model systems for studying synaptic structure and function with super-resolution methods include **(A)** Chromaffin cells **(B)** Hippocampal cell culture **(C)** Retinal biopolar neurons **(D)** *Drosophila* neuromuscular junction **(E)** Calyx of Held **(F)** Isolated mossy fiber boutons. However, all these model systems have their advantages and disadvantages, which we briefly discuss in this figure.

Besides such common features, EM also revealed a remarkable diversity in synaptic ultrastructure both between different organisms and between neuronal types (Figure 1). Synapses at the neuromuscular junction (NMJ) vary noticeably between species but display common structural features, such as their large size compared to CNS synapses (30 μm in mice). NMJs are also notable for their complex internal structure including hundreds of individual regularly distributed AZs with a mean inter-AZ spacing of about 1 μm (Rowley et al., 2007). In vertebrates, the synaptic cleft of the NMJ contains the basal lamina and evidently, the width of the cleft is much larger compared to CNS synapses (de Harven and Coërs, 1959). In the NMJ of *Drosophila* larvae (Figure 1D), CAZ proteins form specialized electron-dense projections, the so called T-bars (Prokop and Meinertzhagen, 2006).

Large specialized hippocampal mossy fiber boutons in the mammalian CNS (Figure 1F) contain more than a few ten AZs and contact multiple postsynaptic partners (Rollenhagen et al., 2007). Another type of specialized synapses comprises ribbon synapses of the visual and auditory system of vertebrates (Figure 1C). Their distinguishing feature is the presence of large rod-shaped structures in the presynaptic terminal. These structures are joined by dozens of SVs (Sjoestrand, 1958). It is believed that these ribbons facilitate fusion of SVs with the presynaptic membrane either by an active mechanism that shuttles SVs downward toward docking/release sites (conveyor belt model) or by compound fusion of SVs allowing multivesicular release and thus, extremely high release rates (safety belt hypothesis) (Parsons and Sterling, 2003; Matthews and Sterling, 2008). Remarkably, presynaptic filamentous structures that partially resemble those of ribbons also exist in the cytomatrix of hippocampal boutons (Siksou et al., 2007).

In classical EM chemical fixation methods are applied, such as aldehyde and/or osmium fixation. Fixation is typically followed by dehydration, embedding in a suitable resin, slicing and heavy metal staining (Hayat, 2000). Large structures can be reconstructed in 3D by manual serial sectioning (Stevens et al., 1980), automated serial block face imaging (Denk and Horstmann, 2004) or by focused ion beam (FIB) milling in combination with scanning electron microscopy (SEM) (Knott et al., 2008). For the 3D reconstruction of fine structures, electron tomography (ET) with axial resolution well below slice thickness can be utilized (Perkins et al., 2015).

However, chemical fixation may alter the synaptic ultrastructure by inducing protein polymerization and tissue shrinkage. For example, EM tomography in frog NMJ revealed intricate scaffold structures forming a highly ordered network. The macromolecular assemblies could be sorted according to their shape into distinct classes such as beams, pegs or booms (Harlow et al., 2001, 2013). Nonetheless, the dense projections observed in these chemically fixed synapses might at least partially stem from condensation and collapse of filamentous structures by cross-linking during chemical fixation. In recent years, physical fixation methods have become widespread, and these methods allow overcoming most of the artifacts inherent in chemical fixation.

High-pressure freezing (HPF) followed by freeze-substitution preserves the synaptic ultrastructure significantly better and avoids distortions of the cellular morphology induced by slow chemical fixation (Studer et al., 2001; Rostaing et al., 2006). In addition, it allows rapid vitrification within a few tens of milliseconds even for thicker specimens. HPF in combination with ET of rat hippocampal slices uncovered that SVs are surrounded by a dense network of filaments that link SVs together. Furthermore, longer filaments were observed that directly link SVs to the AZ (Siksou et al., 2007). Nevertheless, HPF is also not artifact-free since ice-crystal formation, high pressure-induced shearing and collapse of air-filled spaces can deform the tissue.

In freeze fracturing, samples are also rapidly frozen and then broken up in the vacuum. A carbon-platinum replica is prepared from the fractured sample surface that can be analyzed by EM (Moor and Mühlethaler, 1963; Bullivant and Ames, 1966). Since PMs can be split into half-membrane leaflets, this method is very well suited for membrane studies. In addition, epitopes of membrane proteins are partially preserved for immune-gold labeling after SDS replica cleaning (Fujimoto, 1995; Masugi-Tokita et al., 2007). This method contributed to the further characterization of presynaptic SV recycling (Heuser et al., 1979; Heuser and Reese, 1981). However, as with HPF, freeze fracturing necessitates rapid cooling and some biological samples require treatment with a cryo-protectant to minimize ice crystal damage.

Cryo-electron tomography (cryo-ET) in conjunction with cryo-fixation techniques and relative mild sample preparation also aims to overcome some of the above mentioned limitations and allows reconstruction of molecular assemblies under more native conditions (Lucić et al., 2005). This technique is discussed in more detail in a later section.

Though for all EM techniques the preservation of biological structures in a state as native as possible is a crucial and vividly discussed issue, EM in principle resolves fine structures well and in conjunction with correlative approaches, like electrophysiology and genetic modification of synapses by gene ablation, EM gave a wealth of information on the structural organization of synapses. Yet, labeling of specific proteins to unravel the exact molecular assembly of proteins remains challenging in EM. Pre- or post-embedding protein labeling using gold-conjugated antibodies usually results in low labeling densities. A notable exception is the giant reticulospinal axons in lamprey. These axons can be cut along their longitudinal axis providing access for antibodies to target the sites of SV recycling (Evergren et al., 2004).

Genetically encoded tags suitable for EM, like the singlet oxygen generator miniSOG (Shu et al., 2011) or the peroxidase APEX (Martell et al., 2012) are still far from being used routinely since the experimental conditions for the generation of precipitate resolvable by EM have to be carefully adjusted. Thus, we are still lacking tools in EM akin to fluorescent proteins in cell biology and light microscopy.

## Sub-Diffraction Microscopy: Bearing Fruit After a Decade of Implementation

A wide range of fluorescence microscopy techniques using different physical principles to overcome the diffraction limit has emerged during the last two decades (for review see, e.g., Sahl et al., 2017; Schermelleh et al., 2019) and consequently, these techniques found their way into neuroscience (Tønnesen and Nägerl, 2013; Igarashi et al., 2018). Among these super-resolution (SR) concepts, stimulated emission depletion (STED) and stochastic optical reconstruction microscopy (STORM) or photoactivated localization microscopy (PALM) are the most commonly used techniques and have been rewarded with the Noble Prize for Chemistry in 2014. Here, we briefly describe the main principles underlying these techniques (Table 1).

**TABLE 1:** Comparison of different super-resolution methods.

Super-resolution method	SIM	STED	Single molecule localization microscopy		
			STORM	PALM/sptPALM	DNA-PAINT
Illumination/Detection	Wide-field/TIRF	Scanning confocal	Wide-field/TIRF	Wide-field/TIRF	TIRF/spinning disk confocal
Lateral resolution	∼100 nm (linear) ∼60 nm (non-linear)	40–70 nm	10–30 nm	10–30 nm	10–30 nm
Axial resolution	300 nm (3D SIM)	Down to 40 nm (iso-STED)	∼20 nm (astigmatism) ∼10 nm (interference)	∼20 nm (astigmatism) ∼10 nm (interference)	∼100 nm (TIRF) ∼80 nm (astigmatism)
Acquisition time	Seconds	Seconds	Minutes	ms-seconds (sptPALM) minutes (PALM)	Minutes-hours
Dyes	Conventional	Dyes suitable for efficient stimulated emission	Photoswitchable dyes	Photo-activatable fluorescent proteins	Dye-conjugated oligonucleotides
Live cell imaging	Yes	Yes	No	Yes	Very limited
Number of frames for single SR-image	9–15 frames	1 frame	Several thousand frames	Several thousand frames	Several thousand frames
Post-processing	Yes (reconstruction in reciprocal space)	No	Yes (emitter localization)	Yes (emitter localization)	Yes (emitter localization)

In STED microscopy, the effective excitation volume is shrunk by overlaying the excitation spot with a doughnut-shaped red-shifted depletion laser that de-excites molecules in the periphery of the excitation spot. By these means, a resolution of 40 nm and below can be achieved (Hell and Wichmann, 1994; Klar et al., 2000).

**FIGURE 2:**
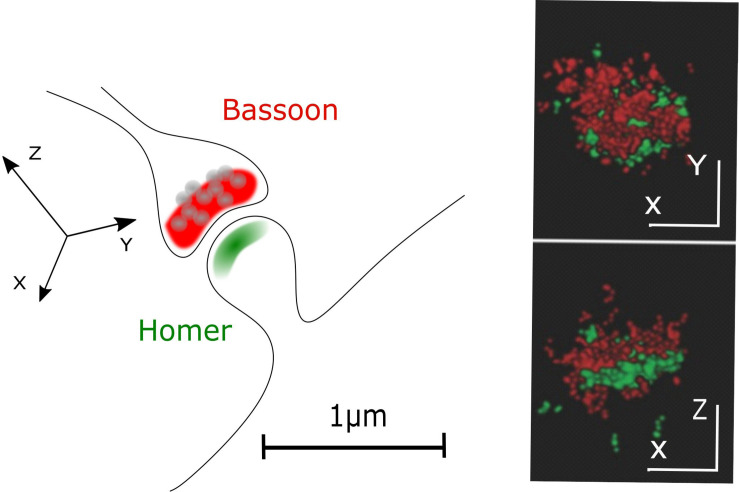
Pre- and postsynaptic compartments resolved with STORM microscopy. A classical CNS synapse and STORM reconstruction of primary hippocampal neurons stained for presynaptic (Bassoon, red) and postsynaptic (Homer, green) markers (adapted from Boening et al., 2017; Copyright (2017) Wiley. Used with permission).

In STORM or PALM, stochastic switching of photoactivatable (PA) or switchable fluorophores is employed to visualize single emitters. Subsequently, the intensity profiles of single emitters are fit by, e.g., a 2D Gaussian function to determine the exact localization of these molecules. After repeatedly imaging different subsets of fluorescent molecules, a high-resolution image is reconstructed by summing up the single molecule coordinates. Depending on the number of detected photons per molecule, a localization precision down to 10 nm is feasible (Betzig et al., 2006; Hess et al., 2006; Bates et al., 2007) allowing to separate, e.g., pre- and postsynaptic compartments with fluorescence light microscopy (Figure 2).

Other concepts in SR microscopy are structured illumination microscopy (SIM) and lattice light sheet microscopy (LLSM). In SIM, the sample is illuminated with a periodic excitation pattern. By these means, high-resolution information is shifted into the resolvable regime and can be extracted by linear image processing to obtain a final image with twofold increased resolution (Gustafsson, 2000). Resolution in SIM can be even further improved by using nonlinear structured illumination patterns (Gustafsson, 2005). LLSM is a specialized version of ultramicroscopy in which light sheets are created by 2D optical lattices. This illumination mode enables high spatiotemporal resolution and low phototoxicity for live cell imaging. Furthermore, LLSM can be operated in different modes allowing either high-speed 3D imaging down to the single molecule level or spatial resolution beyond the diffraction limit (Chen et al., 2014).

A further SR approach that was recently introduced is MINFLUX. Like in STED microscopy, the exact position of individual molecules is determined with a doughnut-shaped laser beam. However, the doughnut is not used to deplete but to excite fluorescence, and emitter positions are probed with the local intensity minimum of the doughnut. This way, the absolute photon number for precise emitter localization is minimized (Balzarotti et al., 2017).

While at first the application of these techniques was reserved for a few expert laboratories, the commercial availability of these instruments today also enables the non-advanced user to perform SR microscopy. Here, we briefly summarize some of the key applications in the neuronal field.

Time-lapse STED microscopy has been successfully employed to visualize plasticity-dependent morphological changes of YFP-positive dendritic spines in hippocampal organotypical slices with a resolution of ∼ 70 nm (Nägerl et al., 2008) and the distribution and dynamics of actin within spines could be resolved at a resolution of 60–80 nm in (Urban et al., 2011).

The distances between synaptic proteins and even individual epitopes of single proteins in fixed brain slices and cultured neurons were determined using 3D STORM (Dani et al., 2010; Herrmannsdörfer et al., 2017; Lagache et al., 2018). These studies revealed, e.g., that the large AZ proteins Bassoon and Piccolo are organized in an extended and oriented manner forming the CAZ together with other presynaptic proteins.

PALM in combination with single particle tracking (sptPALM) was used in live cells to analyze the distribution and mobility of individual synaptic proteins such as Syntaxin1A (Bademosi et al., 2017), voltage gated Ca^2+^ channels (VGCCs) (Schneider et al., 2015; Heck et al., 2019) and postsynaptic AMPA receptors (Hoze et al., 2012; Nair et al., 2013). The results show, e.g., that physical interactions with a large number of PM surface binding sites rather than molecular crowding is responsible for the high density of AMPA receptors at the postsynapse (Hoze et al., 2012).

However, one of the most surprising observations in this context is certainly the discovery of periodic Actin cytoskeleton rings by STORM and STED microscopy (Xu et al., 2013; D’Este et al., 2015). Here it was shown that Actin and Spectrin form alternating ring-like structures with a periodicity of 180–190 nm to stabilize the dendritic and axonal PM.

The application of SR techniques to the presynapse comes along with several challenges. Synapses are usually small structures with a high density of supramolecular complexes. The orientation in space of CNS synapses in culture or slices is random, complicating reconstruction of synaptic structures by simple averaging approaches unless 3D imaging is performed. In addition, the differentiation of pre- and postsynaptic structures is hampered by the small width of the synaptic cleft. Therefore, many structural reconstructions of protein distributions have been performed on large and highly specialized synapses such as the NMJ of *Drosophila* larvae (Figure 1D).

## The Presynaptic Compartment and SV Recycling

Excitatory synapses of cultured hippocampal neurons have a diameter of 600–800 nm and are densely filled with SVs of 40 nm size (Schikorski and Stevens, 1997). A combination of genetic perturbation, electrophysiology, EM and fluorescence microscopy culminated in the current model of NT release (Lisman et al., 2007). First, membrane docked SVs fuse with the presynaptic PM by Ca^2+^-triggered exocytosis. Subsequently, exocytosed SV membranes and proteins are resorted and recycled by triggered compensatory endocytosis, followed by refilling of newly formed SVs with NTs. The hallmark of presynaptic SV recycling is the tight coupling of exo- and endocytosis in space and time, which is necessary to sustain high release rates. Therefore, processes like exocytosis, release site clearance, re-sorting of SV components post fusion and endocytosis have to occur in a highly coordinated manner. A complex set of proteins is required to organize the SV release and retrieval machinery and they are a natural target for SR microscopy to elucidate their molecular organization and dynamics at the presynapse to finally address the following questions: (1) Are there defined SV fusion sites? (2) How tight are release sites and VGCCs coupled in space? (3) Are there defined endocytic sites? (4) What is the mechanism responsible for tight temporal and spatial coupling of exo- and endocytosis? (5) What is the fate of SV proteins at the presynaptic PM after SV fusion? (6) Are presynaptic release sites and postsynaptic NT receptors spatially correlated across the synaptic cleft?

## AZ Architecture and Organization of SV Release Sites

**FIGURE 3:**
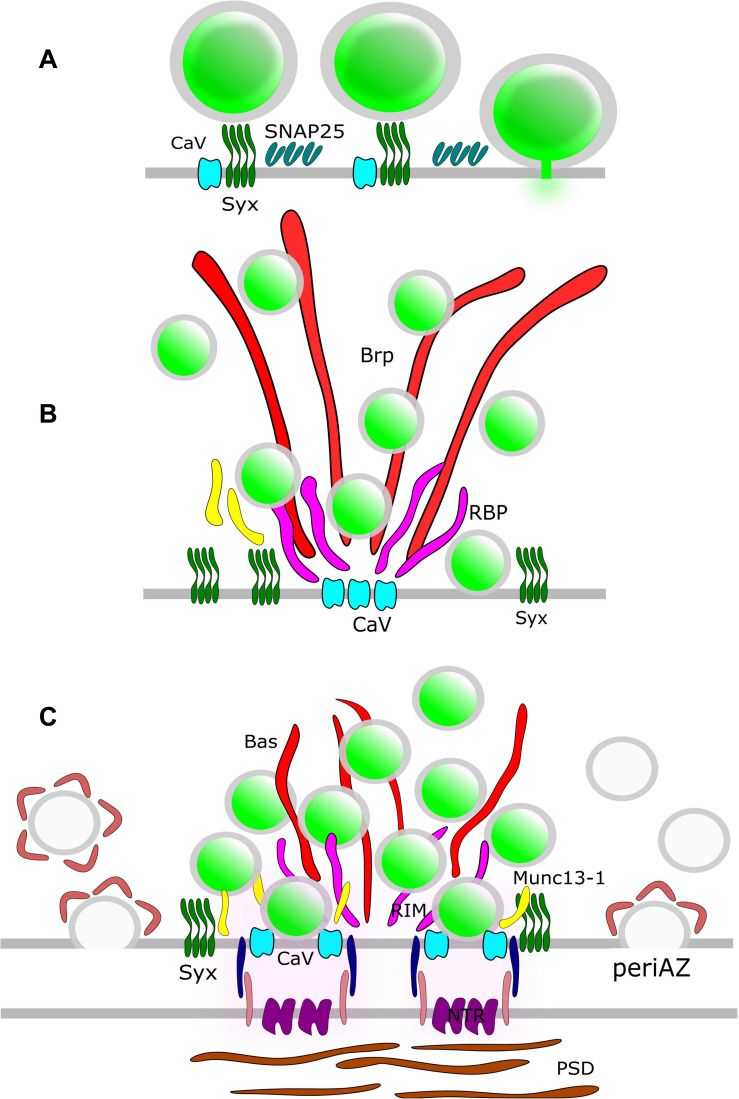
AZ Nano-architecture in common synapse models as suggested by SR microscopy. **(A)** Rat neuroendocrine cells. SR microscopy revealed syntaxin clusters (Syx) potentially serve as docking sites for dense-core vesicles in neuroendocrine cells. Vesicle fusion causes disassembly of the Syx nanoclusters. SNAP25 also form clusters at the plasma membrane; nevertheless, these clusters do not overlap with Syx. **(B)** Drosophila NMJ. The large T-bar forming proteins Bruchpilot (BRP) and Rim-binding protein (RBP) are positioned with their *N*-terminus and *C*-terminus, respectively, close to Ca^2+^-channel clusters (CaV). Syx clusters are situated in the active zone near Brp and RBP. **(C)** Mammalian CNS synapse. The large filamentous protein Bassoon (Bas) faces the AZ with its *C*-terminus. Munc13-1, CaV, and Syx clusters are positioned close to the SV release sites. The postsynaptic neurotransmitter receptors (NTRs) are aligned with SV fusion sites in a columnar manner via synaptic cell adhesion molecules (SCAMs). Compensatory endocytosis of SVs occurs in a spatially distinct region of the presynapse, the peri-active zone. Here, a surface fraction of SV proteins, the readily retrievable pool (RRetP), is pre-assembled.

Synaptic vesicles fuse with the presynaptic PM at the AZ (Heuser et al., 1979). In order to elucidate the molecular organization of the underlying release sites, it is essential to understand the molecular assembly and relative position of CAZ-proteins, VGCCs, SNARE proteins and other release factors. STED microscopy has been intensively used in the NMJ of *Drosophila* larvae to unravel the molecular scaffold responsible for AZ organization (Figure 3). These presynaptic terminals contain several AZs and the protein Bruchpilot (BRP) was observed in doughnut-shaped structures centered at these AZs (Kittel et al., 2006). AZs of a BRP mutant displayed loss of T-bars, reduced clustering of VGCCs and depressed evoked SV release. Thus, BRP was one of the first scaffolding proteins identified as being responsible for AZ integrity by establishing a close proximity between docked SVs and VGCCs. Later on, other CAZ proteins such as Rab3-interacting molecule (RIM), RIM-binding protein (RIM-BP) and Fife were identified to play major roles in correct AZ formation and thus, NT release (Liu et al., 2011; Graf et al., 2012; Bruckner et al., 2017). A recent two-color STED microscopy study in *Drosophila* NMJ uncovered yet another protein, the priming factor Unc13A, as essential for stable release site generation (Reddy-Alla et al., 2017).

The mammalian CAST/ELKs proteins are orthologs of BRP and deletion of CAST/ELKs, similar to deletion of BRP in *Drosophila*, led to impairment of the AZ ultrastructure in mouse retinal ribbon synapses (Hagiwara et al., 2018). In contrast, loss of CAST/ELKs hardly affected AZ integrity in cultured hippocampal neurons. Here, only minor effects on readily releasable pool (RRP) size and Ca^2+^ influx were observed (Liu et al., 2014; Held et al., 2016).

In mammalian neurons, the large scaffolding proteins Bassoon and Piccolo are integral parts of the CAZ (Figure 3). Dual-color STED microscopy in mouse NMJ disclosed non-overlapping punctuate patterns for these presynaptic scaffolding proteins, and it was found that the Bassoon puncta co-localized with P/Q type VGCCs (Nishimune et al., 2016), in agreement with the earlier finding that Bassoon localizes P/Q type VGCCs to the AZ in hippocampal cultures (Davydova et al., 2014). Furthermore, loss of Bassoon impairs recruitment of SVs to release sites in mossy fiber synapses (Hallermann et al., 2010). However, despite the aforementioned phenotypes, loss of function studies for Piccolo and Bassoon have not been shown to affect the AZ ultrastructure in CNS synapses (for review see Gundelfinger et al., 2015) pointing to functional redundancy of CAZ proteins in these synapses. The situation is different for vertebrate sensory synapses. In mice hair cells, STED microscopy upon genetic disruption of Bassoon revealed a lack of ribbons and a reduced number of VGCCs at the AZ (Frank et al., 2010). Deletion of Piccolino, a Piccolo splice variant specifically expressed in sensory ribbon synapses, compromised the synaptic ribbon ultrastructure (Regus-Leidig et al., 2014).

Isoforms of Munc13, the mammalian homolog of Unc13, have also been implicated in organizing SV release sites. Munc13-3 regulates density and localization of VGCCs at the AZ (Kusch et al., 2018). However, the role of Munc13 isoforms in presynaptic VGCC recruitment is still disputed. A direct interaction of, e.g., Munc13-1 and VGCCs has been shown (Calloway et al., 2015) but attributed to control of VGCC function rather than recruitment. 3D STORM revealed that Munc13-1 molecules form multiple supramolecular clusters that serve as independent SV release sites by recruiting Syntaxin1A, one of the target SNARE proteins (t-SNARE) in the presynaptic PM (Sakamoto et al., 2018). In this way, Munc13-1 provides platforms for open Syntaxin1A molecules to generate activated SNARE complexes (Rizo, 2018) that facilitate bridging of PM and SVs.

Not surprisingly, Syntaxin1A has emerged as another candidate for organizing the SV fusion machinery. Much of the investigation into this protein has focused on its tendency to cluster at the PM, triggered by the interaction with PIP2 (Milovanovic et al., 2016). Early evidence from neuroendocrine PC12 cells showed the granule fusion sites are “premarked” with Syntaxin1A clusters (Knowles et al., 2010) and L-type VGCCs expressed in HEK cells exhibit strong co-localization with Syntaxin1A clusters (Bar-On et al., 2012; Sajman et al., 2017). The role of Syntaxin1A clusters as release site organizers is challenged by the lack of overlap with clusters of SNAP25, its t-SNARE partner, in PC12 cells (Bar-On et al., 2012) (Figure 3). Moreover, a significant pool of extrasynaptic t-SNARE-proteins exists that is recruited to the presynapse during stimulation (Maidorn et al., 2019). Furthermore, an increase in Syntaxin1A trapping was observed after treatment with the widely used anesthetic propofol (Bademosi et al., 2018). However, propofol-induced clustering of Syntaxin1A is associated with impaired neurotransmission, which lends itself to the conclusion that clustered Syntaxin1A is excluded from the SV fusion process. Thus, the exact function of these t-SNARE clusters in organizing release sites remains open to debate.

In a recent study using live hippocampal neurons, single SV fusion events were detected using the pH-sensitive fluorescent protein pHluorin fused to the vesicular glutamate transporter vGlut (Maschi and Klyachko, 2017). The individual fusion events were spatially mapped with a precision of ∼30 nm and the results show that mammalian CNS synapses indeed display a dozen of stable SV release sites per bouton. Moreover, the spatial pattern of these fusion sites changed in an activity-dependent manner.

To summarize, presynapses harbor distinct SV fusion sites that are defined by a complex interplay between CAZ proteins, VGCCs, Munc13 and t-SNARES and significant contributions from SR microscopy has helped shed to more light on AZ architecture and organization of SV release sites.

## The Mechanism of SV Exocytosis

Exocytosis of SVs is mediated by SNARE-proteins that drive fusion of the SV membrane with the presynaptic PM. However, the exact mechanism of SNARE-mediated membrane fusion is still under debate. Different models have been proposed and one of these involves priming of docked SVs into a stable hemifused intermediate, in which the inner leaflet of the PM is already fused with the outer leaflet of the SV membrane before cargo release (Kweon et al., 2017). ET has been utilized to reconstruct high-resolution images of docked SVs in different preparations like frog NMJ (Jung, 2019), mice photoreceptors (Zampighi et al., 2011) and thin sections of rat brain (Zampighi et al., 2006). In these studies, hemifused structures at SV/PM contact sites could be frequently observed. However, the results depend heavily on image processing and interpretation and have not been accepted in the field as a strong evidence for a stable hemifused primed state for SVs. In addition, these attempts are limited by the fact that a real population of hemifused SVs might simply be lost during chemical fixation.

Recently, evidence supporting the fusion-through-hemifusion model was reported for fusion of dense core vesicles (DCVs) in live bovine chromaffin cells (Zhao et al., 2016). The authors analyzed the reorganization of the inner PM leaflet lipid PtdIns(4,5)P_2_ before and during DCV fusion using 3D STED microscopy. They could observe hemifused Ω-shaped structures seconds before fusion pore opening with the transition to full fusion or fission depending on a completion between fusion and a Ca^2+^/Dynamin mediated fission mechanism.

Furthermore, STED microscopy enabled the observation of dynamic fusion pore behaviors in neuroendocrine cells (Shin et al., 2018). The results showed a surprisingly large pore size range with varying rates for expansion, constriction and closure (kiss-and-run), critically determining cargo release. The same study found constriction to be mediated by Ca^2+^/Dynamin while expansion was driven by Actin-dependent membrane tension. However, these studies were only possible on bovine chromaffin cells with large secretory granules (up to 400–500 nm in diameter). And while chromaffin cells share essentially the same core exocytosis machinery with neurons, AZ specializations are missing (Neher, 2018). At presynaptic AZs, proteins like, e.g., Rim and RimBP are implicated in docking and priming and confer extra speed and an extra layer of control for exocytosis. But interestingly, similar Ω-shaped profiles can be induced in Lamprey synapses by electrical stimulation after treatment with Actin-depolymerizing drugs (Wen et al., 2016), suggesting that Actin-induced merging of Ω-shaped release intermediates also occurs in neurons.

## Spatial Coupling of Ca^2+^Channels and Release Sites

Synaptic vesicles fusion is tightly coupled to the entry of Ca^2+^ ions into the presynapse. Ca^2+^ influx upon opening of VGCCs triggers SV priming and fusion with the presynaptic PM. Precise timing of transmitter release relative to the arrival of an action potential requires a certain proximity between VGCCs and docked SVs. Typically, presynaptic VGCCs organize into distinct clusters (Kittel et al., 2006; Holderith et al., 2012; Nishimune et al., 2016) and using immuno-gold labeling of SDS-treated freeze fracture replicas, it was found that the number of VGCC clusters matches the number of presynaptic SV docking sites (Miki et al., 2017). However, classical experiments with Ca^2+^ chelators like BAPTA and EGTA have shown that the spatial coupling of Ca^2+^ entry points and Ca^2+^ sensors varies, ranging from 10 to 30 nm in some types of cortical glutamatergic and GABAergic synapses (Bucurenciu et al., 2008; Schmidt et al., 2013) to 100 nm in the mature Calyx of Held (Borst and Sakmann, 1996). Using STED microscopy in *Drosophila* NMJ it has been found, that the topology of docked SVs and VGCCs can be regulated by isoform specific interactions between Unc13 and scaffold proteins like Syd-1, Liprin-α, BRP, and Rim-BP (Böhme et al., 2016). In cerebellar preparations weak synapses exhibited three-fold more VGCCs than strong synapses, but with a five-fold longer coupling distance pointing toward a diverse arrangement of SV/VGCCs even in CNS synapses (Rebola et al., 2019).

The picture of VGGC coupling was further enhanced by using sptPALM on live hippocampal neurons (Schneider et al., 2015). Here it was shown that around 60% of VGCCs are mobile while confined to the presynaptic PM. These data suggest that the fractions of mobile and immobile channels are transient within the AZ and that an interplay between channel density, mobility and Ca^2+^ influx supports Ca^2+^ domain co-operativity to control release probability.

## Presynaptic Endocytic Sites and Coupling of Exo-Endocytosis

Synaptic vesicle exo- and endocytosis in the presynapse are temporally coupled but spatially segregated in different PM domains (Wienisch and Klingauf, 2006). Compensatory endocytosis occurs in the peri-AZ (Roos and Kelly, 1999; Teng et al., 1999), and preformed endocytic patches organized around the AZ could be visualized by iso-STED microscopy (Hua et al., 2011). The main pathway for SV retrieval at the peri-AZ under moderate stimulation conditions was considered to be Clathrin-dependent (Heuser and Reese, 1973; Granseth et al., 2006). However, optogenetics in combination with ultrafast freezing followed by EM analysis revealed that after a single stimulus SVs fuse in the AZ and are directly retrieved by a Clathrin-independent mechanism at the AZ periphery within 50–100 ms (Watanabe et al., 2013). EM, however, only provides snapshots of the presynaptic ultrastructure and in order to finally resolve the mechanism of compensatory endocytosis, it is essential to perform high-resolution live-cell imaging and visualize single SV recycling. Video-rate (28 frames/s) STED microscopy already enabled mapping of the movement and mobility of single SVs in live presynaptic boutons (Westphal et al., 2008). Furthermore, spatially highly resolved tracking of single endocytosed SVs has been performed (Joensuu et al., 2016). Nevertheless, these attempts have not yet brought novel insights into the SV retrieval mechanism.

In addition, the mechanism that tightly couples exo-endocytosis in time is controversial and could not finally be resolved with the help of SR microscopy. It has been reported that Ca^2+^ modulates the time course of endocytosis (Sankaranarayanan and Ryan, 2001; Wu et al., 2009; Leitz and Kavalali, 2011). While in the calyx of Held a Ca^2+^/Calmodulin-dependent mechanism was found to highly stimulate and to initiate all modes of endocytosis (Wu et al., 2009), in hippocampal cultures Ca^2+^ inhibits endocytosis for single APs (Leitz and Kavalali, 2011). However, in most studies Ca^2+^ stimulates endocytosis (Sankaranarayanan and Ryan, 2001; Wu et al., 2009; Wu and Wu, 2014). The exact molecular mechanism how Ca^2+^ can couple SV fusion and retrieval remains elusive, but Calmodulin and myosin light chain kinase are strong candidates (Wu et al., 2009; Yue and Xu, 2014). Recently it has been shown that endocytosis is also triggered upon Ca^2+^-independent exocytosis suggesting that compensatory endocytosis might also be initiated by biophysical changes induced by addition of the SV membrane to the presynaptic PM (Orlando et al., 2019). But this finding does not rule out an important role of Ca^2+^ in compensatory endocytosis.

## The Fate of SV Proteins at the Presynaptic PM After SV Fusion

Synaptic vesicle function relies on a distinct set of proteins present in a defined stoichiometry. The molecular sorting mechanisms for individual SV components during exo-endocytosis, however, remain largely unresolved. In one scenario, SV constituents remain clustered upon fusion and diffuse as a raft-like patch to the peri-AZ, preventing the need for re-sorting prior to endocytosis. Indeed, it was shown using live cell STED microscopy that Synaptotagmin1 remains clustered after SV exocytosis (Willig et al., 2006). In contrast, other reports claim rapid dispersion of SV proteins by diffusion upon exocytosis (Wienisch and Klingauf, 2006; Funahashi et al., 2018) and re-sorting and clustering into patches at the peri-AZ (Hua et al., 2011). In this context the exact role of adaptor proteins like, e.g., AP2, Stonin2 and AP180 in productive cargo clustering at endocytic sites is still not fully understood since knockdown or knockout in neurons often resulted in only minor inhibition of SV retrieval (for review see Gauthier-Kemper et al., 2015). The precise sorting of SV constituents for retrieval is a complex process involving self-assembly and several layers of adaptor protein interactions. Thus, the picture remains far from being complete.

## Trans-Cellular Nano-Alignment Between Presynaptic and Postsynaptic Compartments

While on the presynaptic PM stable SV release sites exist at which SVs fuse and release their NT content, the postsynaptic PM harbors the NT receptors that bind NTs. SR microscopy revealed that postsynaptic receptors and scaffolding proteins are organized in clusters of 70–80 nm size (MacGillavry et al., 2013; Nair et al., 2013). Based on these observations the hypothesis was established that PM nanodomains involved in neurotransmission in pre- and postsynaptic membranes are aligned on both sides of the synaptic cleft (Figure 3). Indeed, a trans-synaptic alignment of RIM1 and PSD95 nanoclusters was visualized by 3D STORM in cultured hippocampal neurons (Tang et al., 2016). In inhibitory synapses, postsynaptic GABA_A_ receptors are strongly associated with presynaptic RIM clusters (Crosby et al., 2019). According to this model, RIM nanoclusters define SV release sites that align opposite to postsynaptic receptor-scaffold ensembles within tens of nanometers creating a functional unit across the synaptic cleft. Recently, trans-synaptic nano-alignment was also observed in the mammalian NMJ (York and Zheng, 2017).

Clustered patterns are also described for several synaptic cell adhesion molecules (SCAMs) including postsynaptic LRRTM2 and presynaptic Neurexin1β, while postsynaptic Neuroligin1 is dispersed in dendritic spines (Chamma et al., 2016). In addition, Neurexin1 nanodomains are dynamically regulated by the matrix metalloproteases ADAM-10. Blocking ADAM-10 mediated Neurexin1 cleavage leads to an increase in cluster size (Trotter et al., 2019). These SCAM clusters are likely to be involved in the trans-synaptic alignment as, e.g., expression of truncated Neuroligin1 disrupts trans-synaptic alignment causing mislocalization of SV fusion sites away from AMPAR clusters (Haas et al., 2018).

## Reconstruction of the Cellular Ultrastructure by Localization Microscopy

For the analysis of protein distributions, localization microscopy techniques (STORM and PALM) feature a unique advantage as these pointillist methods provide single molecule coordinates allowing for comprehensive cluster analysis. Cluster formation at pre- and postsynaptic membranes has been described for CAZ proteins, SNARE proteins, SCAMs and NT receptors. Similar protein clusters were observed not only in neurons but also in neuroendocrine cells (Bar-On et al., 2012). Moreover, the advent of localization microscopy prompted a surge of publications reporting clusters of PM and PM-associated proteins in almost every cell type (Aaron et al., 2012; Itano et al., 2014; Lima et al., 2018) underlining the theory, that nano-clustering of PM proteins is an integral part of the hierarchical organization at the PM (Garcia-Parajo et al., 2014). These results have come to be viewed more critically because the protein clusters observed might be at least partially based on artifacts resulting from poor sample preparation or of inappropriate imaging conditions and reconstruction algorithms (Burgert et al., 2015; Culley et al., 2018).

**FIGURE 4:**
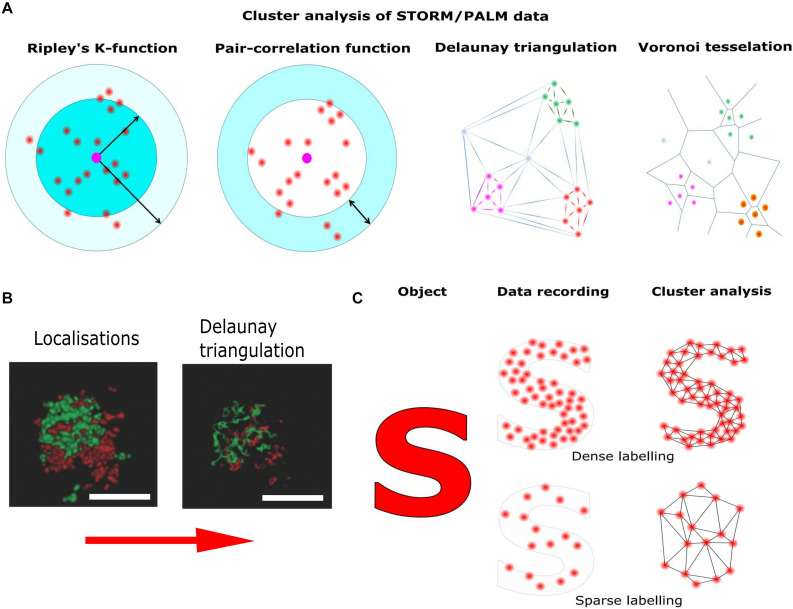
Pointillist localization microscopy and the sparse labeling problem. Localization microscopy techniques base on the precise localization of single emitters followed by image reconstruction using the single molecule coordinates. **(A)** Different methods of cluster analysis for the interpretation of STORM/PALM data. Ripley’s K function describes the molecular density as a function of an area of interest around a reference particle. The radial distribution function (also known as pair correlation function) describes density variations as a function of distance from a reference particle. Delaunay triangulation is a triangulation method such, that no point is inside the circumcircle of any triangle in the triangulation. Delaunay triangulation maximizes the minimum angle of all the angles of the triangles in the triangulation. Cluster distributions can be extracted by deleting edges longer than individually set thresholds. Voronoi tessellation splits the area into convex polygons such that each polygon contains one seed (molecule coordinate). The polygon areas consist of all points that are closer to their generating seed than to any other. Based on individually set thresholds (e.g., molecular density) image segmentation based on the Voronoi diagram results in cluster distributions**. (B)** STORM reconstruction of a hippocampal synapse stained for presynaptic (Bassoon, red) and postsynaptic (Homer, green) markers and Delaunay triangulation built on these localization (adapted from Boening et al., 2017; Copyright (2017) Wiley. Used with permission). **(C)** Though in principle the localization of single emitters can be as precise as 10 nm, the overall resolution is also determined by labeling density. Thus, if the labeling density is not high enough, relevant information is missing and cellular structures are falsely reconstructed.

Aggregate-forming labels and low labeling densities result in apparent protein clusters, which hardly reflect the underlying protein distribution (Figure 4). In addition, artificial clustering can be induced by the interaction of cell membranes with the polymer coating on the cover slide (Santos et al., 2018) or by sample preparation, e.g., during chemical fixation (Whelan and Bell, 2015). Thus, the development of improved labeling strategies is crucial to elucidate structure and function of sub-synaptic compartments.

High labeling densities carries its own risks since, in combination with inappropriate photo-switching rates, it can lead to overlapping single fluorophore signatures (Burgert et al., 2015). This introduces artificial sharpening during data analysis and thus, false protein clusters. This can be overcome with the judicious application of experimental and analytical tools such as variation of labeling density (Baumgart et al., 2016), temporal band pass filtering using Haar wavelet kernels (Marsh et al., 2018) or compressed sensing (Zhu et al., 2012).

Thus, the interpretation of localization microscopy data is a non-trivial task. The localization precision for single molecules scales with the inverse of the square root of the number of detected photons (Thompson et al., 2002) and benchmark studies have been performed to compare different software packages in terms of filtering and fitting algorithms for optimized data analysis (Sage et al., 2015). Nevertheless, the resolution in reconstructed images depends on both, the localization uncertainty and density of fluorescent labels, and several approaches have been proposed to estimate the true resolution in reconstructed images based, e.g., on estimation theory (Fitzgerald et al., 2012) or Fourier ring correlation (Nieuwenhuizen et al., 2013).

Once the single molecule coordinates have been accurately determined, cluster analysis can be applied. For this purpose, several standardized quantitative methods have been proposed. The most widespread methods to distinguish a clustered from a random distribution are nearest neighbor and pair correlation analysis (Sengupta et al., 2011; Rajappa et al., 2016; Sajman et al., 2017). Unfortunately, these methods are susceptible to the level of single molecule background and cluster shape (Lagache et al., 2013). It is possible to detect false clustering caused by stochastic local density increases for proteins mostly uniformly present in the PM (Baumgart et al., 2016). Recently, more advanced methods for cluster analysis have been introduced. These methods avoid artifacts caused by the geometry of the cell surface, the level of protein labeling and multiple blinks of fluorophores (Itano et al., 2014; Dinic et al., 2015). Bayesian statistical analysis of localization data, e.g., significantly increases the chance to find real protein clusters (Rubin-Delanchy et al., 2015; Griffié et al., 2016). However, the most promising methods for cluster analysis are Voronoi tessellation and Delaunay triangulation (Levet et al., 2015; Alán et al., 2016; Andronov et al., 2016; Boening et al., 2017). These methods are only minimally sensitive to the background signals, and are applicable for detecting clusters of various shape (Figure 4).

In addition, PALM exploits the possibility to count the number of individual molecules (Specht et al., 2013). In contrast to small organic fluorophores used in STORM, PA proteins emit a limited number of photons after activation before they irreversibly photobleach. However, most of the PA proteins display a blinking behavior, which may cause an over counting of the molecules in the sample. But knowing the time between multiple appearances of a fluorophore, one can convert the number of detections into the number of molecules (Lee et al., 2012; Nino et al., 2017). Nevertheless, it should be borne in mind that the labeled protein is often expressed in addition to the endogenous protein and thus, the number of molecules in a cluster can be easily over- or underestimated.

## Mobility Analysis by Single Tracking and sptPALM

The era of SR microscopy started with the advent of single molecule imaging and tracking techniques (Schmidt et al., 1996; Dickson et al., 1997; Schütz et al., 2000; Triller and Choquet, 2008; Kusumi et al., 2014). The evolution of these techniques and the fluorescent probes used, from sparse antibody or quantum dot labeling to sptPALM using photo-convertible fluorescent proteins is well documented in a number of studies analyzing localization and mobility of postsynaptic AMPA receptors (Tardin et al., 2003; Opazo et al., 2010; Nair et al., 2013). Tracking the motion of individual protein molecules provides information, which can be used for quantification of molecular mobility (Manley et al., 2008). However, localization precision can be compromised by motion blur, i.e., fast diffusing molecules spread out their emitted photons over multiple pixels. Furthermore, high particle densities can lead to tracking errors when molecules are falsely connected into trajectories. Technical and analytical solutions have been provided that overcome these biases like stroboscobic illumination (Elf et al., 2007) or data analysis using sophisticated kinetic frameworks (Hansen et al., 2018).

sptPALM revealed, that synaptic molecules like VGCCs, Syntaxin1A and AMPA receptors and are highly mobile and usually exhibit only transient trapping in nanodomains (Bademosi et al., 2017; Lee et al., 2017). The relevance of this mobility becomes increasingly clear since it was recently shown that transient confinement of VGCCs shapes presynaptic short term plasticity (Heck et al., 2019) and that AMPA receptor surface diffusion is required for postsynaptic long term potentiation (Penn et al., 2017).

However, in order to gain insights into molecular motion from single molecule trajectories, adequate physical models are needed (Masson et al., 2014; Holcman et al., 2015). Diffusing molecules in the PM often exhibit non-Brownian motion due to interaction with other molecules in the PM, cytoskeletal elements or adaptor proteins (Weigel et al., 2011; Metzler et al., 2016). Hidden Markov chain modeling (HMM) has been applied to distinguish diffusional states with different diffusion coefficients in sptPALM data sets (Persson et al., 2013; Slator and Burroughs, 2018). This kind of analysis not only provides a quantitative description of different diffusion modalities but also an estimate of transition probabilities between them. Such an approach allows different diffusive states to be characterized for Syntaxin1A at the presynaptic PM of *Drosophila* NMJ (Bademosi et al., 2017) and for single SVs which were labeled with internalized fluorescent Vamp2 in hippocampal boutons (Joensuu et al., 2016).

A comprehensive analysis of diffusional properties of proteins in the cell is essential for understanding the molecular underpinnings of cellular processes. However, we cannot measure the diffusion of any protein molecule without the addition of a molecular tag. While fusion of the protein of interest with a fluorescent protein has mostly only a minor effect on mobility (the diffusion coefficient scales (hydrodynamic radius)^–1^ or (molecular mass)^–3^ for spherical molecules according to Stokes’s law) fluorescent proteins may induce artificial dimerization, as reported for EGFP (Snapp et al., 2003). In the PM where the local concentration of PM proteins can be very high due to crowding, dimerization may have a significant impact on protein mobility. It was, e.g., shown that mEos2 causes artificial clustering of PM proteins in the cell (Zhang et al., 2012), putting the diffusion coefficients measured for Syntaxin1A with mEos2 (Bademosi et al., 2017) or anti-EGFPF antibodies (Wang et al., 2014) under debate. Therefore, the use of fluorescent proteins with highly reduced dimerization properties such as Dendra2 or mEos3.2 is preferable for cluster analysis and diffusion coefficient estimation.

## Discussion

What is the status after more than a decade of SR microscopy? At first glance, the wealth of data is impressive. With the help of SR microscopy the architecture of the AZ could be described in more detail, SV fusion sites could be mapped and a trans-synaptic alignment between presynaptic SV fusion sites and postsynaptic NT receptors could be observed. Moreover, due to the applicability of SR microscopy to living cells, it was possible to analyze the single molecule dynamics of presynaptic membrane proteins. On closer examination, however, it is noticeable that structural reconstruction in the range 100–200 nm works well, such as in the case of the periodicity of actin filaments of 190 nm, the width of spine necks or the localization of BRP in doughnut-shaped structures of 190 nm length. However, the fundamental resolution of the corresponding techniques is significantly higher and can be as good as 20–40 nm. Structural reconstructions in this resolution range usually produce an analysis-dependent dot pattern, the meaning of which is subject to an individual interpretation. The outcome strongly depends on the algorithms and thresholds used. The mean cluster size of Syntaxin1A in the PM of PC12 cells, e.g., has been estimated to be around 90 nm by STORM but to be around 60 nm by STED (Sieber et al., 2007; Bar-On et al., 2012). Thus, meticulous acquisition and analysis is required. Fortunately, the toolbox for data acquisition and evaluation is constantly growing, which helps to increase the reliability of SR microscopy data interpretation.

However, in SR microscopy we now face a problem, which has been discussed analogously in EM since years: What is the best labeling procedure to analyze the cellular ultrastructure in a state as native as possible? It has become increasingly obvious that immuno-labeling is subject to a sparse labeling problem, not as strongly as in immuno-gold EM, but in dense protein assemblies like CAZ and PSD this is a severe limitation. Despite this, STORM studies persist in utilizing classic standard labeling methods like immunostaining using primary and secondary antibodies. In principle, promising alternatives are already available such as the use of nanobodies (Ries et al., 2012; Seitz and Rizzoli, 2019) or RNA-based aptamers (Gomes de Castro et al., 2017). Nevertheless, the available labeling strategies are the limiting factor to exploit the full resolution capability of SR microscopy right now.

For other issues like multiplexing, i.e., imaging several proteins of interest, minimizing photon count (and thus photodamaging), and the problem of thick specimens promising solutions have been developed. Most of the variations of STORM, PALM, and STED techniques are not applicable to the use of more than two different fluorescent markers. Recently, DNA-PAINT was introduced which is theoretically unlimited with respect to the number of probes being analyzed (Jungmann et al., 2010; Agasti et al., 2017). Thus, DNA-PAINT appears advantageous for multiplexing and multi-channel reconstruction. However, DNA-PAINT can be best applied in conjunction with total internal refection microscopy (TIRFM), since then the background due to freely diffusing labeled DNA strands is low compared to the signal of bound labeled DNA. Thus, to decipher molecular events in presynaptic boutons with this technique a TIRFM-amenable presynaptic preparation is desirable. Some modifications of dSTORM, such as the recently invented MadSTORM (Yi et al., 2016) are also applicable for multiplexing.

For minimizing the number of detected photons needed for localization and thus avoiding photobleaching as well as photodamage, the recently introduced MINFLUX concept is one of the most promising developments with regard to driving the field forward (Balzarotti et al., 2017). Recently, the postsynaptic protein PSD-95 was imaged with 3D resolution of 2–3 nm in hippocampal cultures using MINFLUX (Gwosch et al., 2020).

For imaging of whole cells and thicker specimens like, e.g., slice preparations, light sheet-based methods, in particular LLSM, will be of great importance in the future since these methods allow imaging with strongly reduced phototoxicity (Dodt et al., 2007; Chen et al., 2014). Bessel lightsheet microscopy has recently successfully combined with imaging of spontaneous blinking fluorophores to obtain an imaging speed of 2.7 × 10^4^ μm^3^ s^–1^ with a lateral resolution of 75 nm (Lu et al., 2019).

Strong limitations for the application of SR methods *in vivo* are high levels of autofluorescence and tissue photodamage, in particular for the green/yellow spectral range (König, 2000; Berning et al., 2012). To overcome these limitations, far red-emitting fluorescent proteins have been employed in STED microscopy (Wegner et al., 2017). However, red-emitting fluorescent proteins display low photostability and quantum yield compared to their shorter wavelengths analogs. Nevertheless, the quality of the *in vivo* SR imaging could be increased by using red and far-red emitting organic fluorophores linked to the protein of interest via click-chemistry (Masch et al., 2018). Here the authors succeeded in recognizing PSD95 domains labeled with the far-red emitting fluorophore SiR in live mouse brain using HaloTag based labeling.

For sptPALM, it will become more relevant to circumvent overexpression of tagged proteins and observe localization and mobility of the endogenous population of proteins. A toolbox for targeted genomic integration of fluorescent tags via CRISPR mediated knock-in in neurons has recently been published (Willems et al., 2019). Additionally, the MINFLUX concept also appears to be an encouraging approach for single molecule tracking since the spatiotemporal resolution can be greatly improved (Eilers et al., 2018).

But despite the enormous achievements of EM and constant improvements in the field of SR fluorescent microscopy, as well as long-term biochemical and electrophysiological studies, many questions regarding synaptic structure and function remain unresolved. The fine structure of the presynaptic AZ and the distribution and function of CAZ-proteins is still not fully understood. The importance of molecular clusters, like e.g., observed for t-SNAREs, remains enigmatic. In addition, the dynamics and molecular mechanisms of exo-endocytosis coupling, compensatory endocytosis and cargo sorting prior to endocytosis are still only incompletely described.

In summary, we conclude that SR microscopy on the one hand did deliver important insights into presynaptic molecular mechanisms and the underlying ultrastructure, on the other hand, SR microscopy could not fully hold its promise. However, the limitations are mostly not the microscopy techniques themselves but lie in sample preparation and labeling strategies. Thus, the development of artifact-free methods for labeling and analysis is still paramount with urgent imperative to the solution of the sparse labeling problem.

What could be the avenues for future structural research? In CNS synapses, however, to date EM has been only combined with immuno-gold labeling to introduce protein-specific contrast. For instance, the regularly spaced cone shaped structures frequently observed in EM at the presynaptic PM (also referred to as dense projections), could be positively correlated with the abundance of the scaffold proteins Piccolo and Bassoon (Limbach et al., 2011). Nevertheless, these probes were chemically fixed, which might introduce artifacts, and immune-gold labeling is in general poor because of steric hindrance (typically 10 nm gold particles are used).

Therefore, correlative light and electron microscopy (CLEM) seems to be a favorable way to go. Here, high-resolution light microscopy provides specific protein distributions while EM unravels the underlying cellular structures. Thus, CLEM mitigates the sparse labeling problem, as structural information is not solely extracted from light microscopy data. This approach has been successfully employed to show that endocytic proteins distribute into distinct spatial zones in relation to the edge of the clathrin lattice in non-neuronal cells using SEM on unroofed cells (Sochacki et al., 2017).

In terms of structure conservation, cryo-ET conjunction with cryo-fixation techniques appears to be superior among EM techniques. Indeed, using cryo-ET on isolated synaptosomes, the dense projections seen in chemically fixed samples (Harlow et al., 2001) could no longer be observed. Instead, numerous small filamentous tethers that link docked SVs to the presynaptic PM could be resolved and it appears that Rim1α plays a critical role in correct tether formation (Fernández-Busnadiego et al., 2013). Recently, an experimental pipeline for CLEM that combines cryo-ET with cryo-fluorescence microscopy has been published (Tao et al., 2018). Here, intact excitatory and inhibitory synapses could be distinguished in hippocampal culture, and their organelles and macromolecules could be visualized close to the native state.

In addition, cryogenic techniques were successfully applied to localization microscopy (Li et al., 2015; Weisenburger et al., 2017). It was shown that fluorophores under cryogenic conditions are much more photostable allowing the collection of more than 10^6^ photons, thus providing down to Ångstrom resolution. Therefore, correlation of cryogenic localization microscopy with cryogenic EM tomography appears to be a promising approach. Recently a platform for correlative 3D imaging of entirely frozen cells using cryo-SR fluorescence microscopy and cryo-FIB EM has been published (Hoffman et al., 2020). However, at the moment such correlative approaches are reserved for a few expert labs and need to be optimized for routine use.

In summary, we conclude that high-resolution microscopy on its own has not fully lived up to its promises, and that we still need to rely on EM. This is likely to remain the case until correlative methods come to full fruition and the development of a “GFP” for EM, i.e., a reliable protein-specific tag for EM, remains elusive.

## Author Contributions

GN, MK, and JK wrote the manuscript. All authors contributed to the article and approved the submitted version.

## Conflict of Interest

The authors declare that the research was conducted in the absence of any commercial or financial relationships that could be construed as a potential conflict of interest.
